# First Report of New Delhi Metallo-β-Lactamase Carbapenemase–Producing *Acinetobacter baumannii* in Peru

**DOI:** 10.4269/ajtmh.18-0802

**Published:** 2019-01-21

**Authors:** Claudio Rocha, Manuela Bernal, Enrique Canal, Paul Rios, Rina Meza, Miguel Lopez, Rosa Burga, Ricardo Abadie, Melita Pizango, Elia Diaz, Alexander Briones, Cesar Ramal-Asayag, William Vicente, James Regeimbal, Andrea McCoy

**Affiliations:** 1 U. S. Naval Medical Research Unit No 6, Lima, Peru;; 2 Hospital Regional de Loreto, Loreto, Peru;; 3 Universidad Nacional de la Amazonia Peruana, Loreto, Peru;; 4 Instituto Nacional de Enfermedades Neoplasicas, Lima, Peru

## Abstract

Here we report the first incidence of New Delhi metallo-β-lactamase (NDM-1)–producing *Acinetobacter baumannii* in Peru, identified via a strain-based nosocomial surveillance project carried out in Lima and Iquitos. The *bla*_NDM-1_ gene was detected by multiplex polymerase chain reaction (PCR) and confirmed by loci sequencing. *Acinetobacter baumannii* is a nearly ubiquitous and promiscuous nosocomial pathogen, and the acquisition of *bla*_NDM-1_ by *A. baumannii* may facilitate an increase in the prevalence of this important resistance marker in other nosocomial pathogens.

## INTRODUCTION

Health-care–associated infections (HAI) and antimicrobial resistance are of great public health concern worldwide.^1^ Bacterial pathogens causing HAI have become increasingly more resistant over the past 10–15 years as a result of various mechanisms, including gene-mediated enzymes such as class A, B, and D β-lactamases.^2^ Numerous factors, from the horizontal transfer of gene-encoded enzymes to global travel, have allowed resistance and resistant organisms to rapidly spread with great clinical and epidemiological impact.^3,4^ The recently described carbapenemase, New Delhi metallo-β-lactamase (NDM-1) class B, is of particular epidemiological and clinical concern. Since its discovery in 2008, NDM-1 has rapidly spread worldwide and confers resistance to almost all lactams, with the exception of aztreonam, leaving limited therapeutic options against pathogens harboring NDM-1, typically colistin, tigecycline, and fosfomycin.^2,5,6^ In Latin America, the *bla*_NDM-1_ gene was first reported in 2011 from Guatemala and Colombia, and later from Mexico in 2012, Brazil in 2013, and Uruguay in 2013, with all instances from Enterobacteriaceae.^5^ The *bla*_NDM-1_ gene in non-fermentative pathogens was first reported in Latin America from Honduras (*Acinetobacter baumannii*) and Paraguay (*Acinetobacter pittii*) in 2012, and later from Brazil (*A. baumannii*) in 2014, Cuba (*Acinetobacter soli*) in 2015, Argentina (*Acinetobacter junii*) in 2016, and Colombia (*A. baumannii*) in 2017.^5–8^ In Peru, the first report of *bla*_NDM-1_ was in May 2017 in a set of nine *Klebsiella pneumoniae* infecting or colonizing critically ill neurological patients from one hospital in Lima.^9^ Here, we describe the identification of the first three strains of *A. baumannii* harboring *bla*_NDM-1_ in Peru as part of a strain-based surveillance project carried out by the Naval Medical Research Unit No Six (NAMRU-6) in Lima, Peru.

## MATERIALS AND METHODS

In June 2016, NAMRU-6 began ongoing strain collection from Lima (three hospitals) and Iquitos (two hospitals), Peru. Strain collection for this effort focuses on, but is not limited to, multidrug-resistant or extremely drug-resistant (MDR/XDR) ESKAPE pathogens (*Enterococcus faecium*, *Staphylococcus aureus*, *K. pneumoniae*, *A. baumannii*, *Pseudomonas aeruginosa*, and *Enterobacter* spp.), described as the most important pathogens causing HAI globally.^10^

At participating hospitals in Lima and Iquitos, clinicians order sampling and cultures as part of routine patient care (hemoculture, wound swabs, secretion sampling, catheter tip cultures, etc.), and the local hospital laboratory or infection control program isolates and identifies the etiology of the infection, as well as performs susceptibility testing. Briefly, at both sites, conventional culture procedures are performed for identification and susceptibility testing using routine biochemical algorithms and disk diffusion tests, respectively. In addition, at the participating hospital in Lima, isolates from bacteremic patients were recovered using the automated BD BACTEC blood culture system and identification and susceptibilities were confirmed using a Phoenix system (Becton & Dickinson Diagnostics, Sparks, MD).

Once isolates are identified at the participating hospital laboratory, the strains are cryopreserved and transferred within 2 weeks to NAMRU-6 for confirmation and further molecular characterization. No clinical, epidemiological, or personally identifiable data are obtained by NAMRU-6; only limited data about how a strain was isolated are reported.

At NAMRU-6, all strains such as those reported here are confirmed by routine biochemical algorithms and matrix-assisted laser desorption/ionization-time-of-flight (MALDI-TOF) (Becton & Dickinson Bruker Microflex, Bremen, Germany). Antibiotic susceptibility testing (AST) is performed by using the disk diffusion test and the Phoenix system (BD Diagnostics, Sparks, MD), and results are interpreted using the Clinical Laboratory Standards Institute guidelines.^11^ Molecular characterization of strains is performed on highly resistant MDR/XDR pathogens as described by Magiorakos et al.^12^ Each MDR/XDR isolate is first characterized by multiplex PCR. Colony PCR and cycling conditions are performed essentially as described by Poirel et al.,^13^ using the following primers that amplify loci within each open reading frame as described previously^13,14^:

IMP-F: 5′-GGAATAGAGTGGCTTAAYTCTC-3′, IMP-R: 3′-GTTTAAYAAAACAACCACC-5′ (232 bp); VIM-F: 5′-GATGGTGTTTGGTCGCATA-3′, VIM-R: 3′-CGAATGCGCAGCACCAG-5′ (390 bp); NDM-F: 5′-GGTTTGGCGATCTGGTTTTC-3′, NDM-R: 3′-CGGAATGGCTCATCACGATC-5′(621 bp); and KPC-F: 5′-CGTCTAGTTCTGCTGTCTTG-3′, KPC-R: 3′-CTTGTCATCCTTGTTAGGCG-5′ (798 bp).

Cleaned colony PCR products from each PCR-positive isolate are then subjected to amplicon sequencing using an ABI 3130XL genetic analyzer (Applied Biosystems, New York, NY) according to the manufacturer’s protocol, using the same primers listed previously. Sequence analysis is performed using Sequencher V5.4 Gene Codes Corporation (Ann Arbor, MI) and basic local alignment search tool (BLAST, National Center for Biotechnology Information) to compare obtained target sequences with the database. Thus, MDR/XDR strains are characterized by NAMRU-6 for phenotypic and genotypic resistance to the partial sequence level.

## RESULTS

From June 2016 to November 2017, 875 ESKAPE and 213 *Escherichia coli* isolates were collected from Lima and Iquitos hospitals (Table 1). The most frequent pathogen collected from Lima was *P. aeruginosa* (36%), whereas that of Iquitos was *E. coli* (46%). Enterobacteriaceae (*K. pneumoniae*, *Enterobacter* spp., and *E. coli*) represented 45% of all pathogens collected, whereas *A. baumannii* represented 11%. From the total 1,088 strains collected, 978 were Gram-negative pathogens and AST was performed on 974. Of the 974 isolates, 472 (48%) were non-susceptible (resistant or intermediate phenotype) to carbapenem. PCR was performed on 764 (78%) of the 974 strains with AST results, and 155 (20%) of those were positive for at least one of the four carbapenemase-encoding genes tested. Interestingly, 8 (5%) strains harboring PCR-detectable carbapenemase-encoding genes were susceptible to carbapenems, indicating not only a general agreement between PCR and AST but also that there are pseudogenes for carbapenem resistance present in the susceptible clinical isolates here. Such sequences likely have an interesting role in the dynamics of resistance within bacterial populations, and further analysis of such sequences in populations of clinical isolates in Peru is needed. From these 155 strains, an additional eight non-susceptible strains harboring *bla*_NDM-1_ were detected: five Enterobacteriaceae (one *E. coli* and four *K. pneumoniae*, all from Lima) and three *A. baumannii* (one from Lima and two from Iquitos).

**Table 1:** ESKAPE strains and *Escherichia coli* collected from June 2016 to November 2017 from Lima and Iquitos, Peru

ESKAPE	Lima	Iquitos	Total
*Enterococcus faecium*	20	0	20 (1.8%)
*Enterobacter* spp.	38	2	40 (3.7%)
*Staphylococcus aureus*	80	10	90 (8.3%)
*Acinetobacter baumannii*	116	8	124 (11.4%)
*Klebsiella pneumoniae*	204	34	238 (21.9%)
*Pseudomonas aeruginosa*	343	20	363 (33.4%)
Total (ESKAPE)	801	74	875 (80.4%)
*E. coli*	150	63	213 (19.6%)
Total (ESKAPE and *E. coli*)	951	137	1,088

ESKAPE = *Enterobacter* spp., *S. aureus*, *K. pneumoniae*, *A. baumannii*, *P. aeruginosa*, and *E. faecium*.

The only *A. baumannii* harboring *bla*_NDM-1_ from Lima was isolated in January 2017 from a hemoculture taken in a tertiary care oncology hospital and was classified as MDR using the same criteria as previously mentioned.^12^ The strain was resistant to all β-lactams with the exception of aztreonam, to which it showed intermediate resistance. This strain was also resistant to all the carbapenems tested, as well as to fosfomycin. In addition, the strain showed intermediate resistance to tetracycline but was sensitive to quinolones, aminoglycosides, trimethoprim–sulfamethoxazole, and colistin (Table 2). The two *A. baumannii* strains from Iquitos harboring *bla*_NDM-1_ were isolated from the same tertiary care general hospital but at different times and from different types of infections. The first was also isolated in January 2017 from a central venous catheter tip and was classified as MDR. This strain was resistant to all β-lactams with the exception of piperacillin–tazobactam, to which it showed intermediate resistance, and it was also resistant to all the carbapenems tested and fosfomycin. In addition, this strain also showed intermediate resistance to ciprofloxacin but was sensitive to tetracycline, trimethoprim–sulfamethoxazole, levofloxacin, aminoglycosides, and colistin. The second *A. baumannii* from Iquitos was isolated in February 2017 from a postsurgical wound and was classified as XDR. This strain was resistant to all the antibiotics tested except ampicillin–sulbactam, to which it showed intermediate resistance, and colistin and tetracycline, to which it was sensitive (Table 2). Amplicon sequencing of all three *A. baumannii* strains returned a 99% match to *bla*_NDM-1_.

**Table 2:** Resistance pattern of the three strains of *Acinetobacter baumannii* harboring *bla*_NDM-1_ gene

Code	NSC2058	NSI1409	NSI1428
Study site	Lima	Iquitos	Iquitos
Sample type	Hemoculture	CVC tip	Wound
Sample date	January 27, 2017	January 18, 2017	January 17, 2017
AMK	S	S	R
GEN	S	S	R
AMP	R	R	R
SAM	R	R	I
TIC	R	R	R
TIM	R	R	R
TZP	R	I	R
ATM	I	R	R
CAZ	R	R	R
CFZ	R	R	R
CRO	R	R	R
FOX	R	R	R
FEP	R	R	R
LVX	S	S	R
CIP	S	I	R
IPM	R	R	R
MEM	R	R	R
FOF	R	R	R
SXT	S	S	R
TET	I	S	S
CST	S	S	S
Definition for acquired resistance	MDR	MDR	XDR

AMK = amikacin; AMP = ampicillin; ATM = aztreonam; CAZ = ceftazidime; CFZ = cefazolin; CIP = ciprofloxacin; CRO = ceftriaxone; CST = colistin; CVC = central venous catheter; FEP = cefepime; FOF = fosfomycin; FOX = cefoxitin; GEN = gentamicin; I = intermediate; IPM = imipenem; LVX = levofloxacin; MDR = multidrug resistant; MEM = meropenem; NDM-1= New Delhi metallo-β-lactamase; R = resistant; S = sensitive; SAM = ampicillin–sulbactam; SXT = trimethoprim–sulfamethoxazole; TET = tetracycline; TIC = ticarcillin; TIM = ticarcillin-clavulanic acid; TZP = piperacillin–tazobactam; XDR = extremely drug resistant.

## DISCUSSION

Carbapenem-resistant *A. baumannii* is a serious public health concern worldwide.^2,6,15,16^ *Acinetobacter baumannii* has spread throughout the world acquiring antibiotic resistance, first to the most common antibiotics (β-lactams, sulfonamides, and aminoglycosides) and later to carbapenems.^15,17,18^ The clinical impact of this pathogen stems from its ability to upregulate or readily acquire resistance determinants and survive for prolonged periods in nosocomial environments, thus enhancing its rapid dissemination.^6,16,17^ Carbapenem-resistant *A. baumannii* is mainly thought to produce OXA-β-lactamases (oxacillin-hydrolysing) and is less frequently an NDM-1 producer.^15–17^ Conversely, some studies have found truncated forms of the *bla*_NDM-1_-harboring composite transposons Tn*125* in Enterobacteriaceae species, whereas the complete sequence can be found in *A. baumannii*. Taken together, this suggests that *A. baumannii* may serve as a reservoir of *bla*_NDM-1_ that can be transferred to other pathogens.^2,6,19,20^ Furthermore, the spread of *A. baumannii* harboring *bla*_NDM-1_ is likely not a matter of a single lineage but may involve different contemporary clones.

NSC2058 and NSI1409 were found in Lima and Iquitos, respectively; yet they have similar patterns of resistance for the antibiotics tested (Table 2), and both were found in January 2017. Together these results suggest a possible epidemiological link, despite the fact that Iquitos can only be reached by air or river travel and is located more than 1,000 km northeast of Lima. Conversely, NSI1428 was found almost a month later in Iquitos and has a different resistance pattern, suggesting this may be a different clone. However, more sequence analysis beyond the scope here would be required to assess the relatedness of these strains.

We report here the first instances of *bla*_NDM-1_ in *A. baumannii* in Peru, isolated from two different and geographically distinct regions at essentially the same time. This finding highlights the urgent need to implement surveillance and infection control measures for carbapenem-resistant pathogens, not only to mitigate the spread of resistant strains but also to limit or prevent the transfer of these resistance markers between pathogens in Peru and globally.^1^
